# Bivalirudin Presents a Favorable Safety Profile Regarding Adverse Drug Reactions, Thrombocytopenia, and Bleeding in Chinese Patients With High Bleeding Risk Undergoing Percutaneous Coronary Intervention: A Prospective, Multi-Center, Intensive Monitoring Study

**DOI:** 10.3389/fcvm.2022.821322

**Published:** 2022-06-16

**Authors:** Xiaoping Peng, Zhenyong Li, Dunheng Li, Zhongyin Li, Zhaohua Lu, Caidong Luo, Zheng Ji

**Affiliations:** ^1^Department of Cardiovascular, The First Affiliated Hospital of Nanchang University, Nanchang, China; ^2^Department of Cardiology, Xuzhou Central Hospital, Xuzhou, China; ^3^Department of Cardiology, Tai’an First People’s Hospital, Tai’an, China; ^4^Department of Cardiovascular, Puyang Oilfield General Hospital, Puyang, China; ^5^Department of Cardiology, Wuzhou People’s Hospital, Wuzhou, China; ^6^Department of Cardiology, Mianyang Central Hospital, Mianyang, China; ^7^First Department of Cardiology, Tangshan Worker’s Hospital, Tangshan, China

**Keywords:** bivalirudin, percutaneous coronary intervention, high bleeding risk patients, adverse events and adverse drug reactions, thrombocytopenia and bleeding

## Abstract

**Background:**

This study aimed to comprehensively explore the occurrence and risk factors for adverse events (AEs) and adverse drug reactions (ADRs) (especially for thrombocytopenia and bleeding) in Chinese patients with high bleeding risk (older adults, or complicated with diabetes mellitus or renal function impairment) undergoing percutaneous coronary intervention (PCI) with bivalirudin as an anticoagulant.

**Methods:**

A total of 1,226 patients with high bleeding risk who received PCI with bivalirudin as an anticoagulant from 27 Chinese medical centers were enrolled in this prospective, multi-center, intensive monitoring study. AEs, ADRs, thrombocytopenia, and bleeding were collected from admission to 72 h post-bivalirudin administration; subsequently, patients were followed up on the 30th day with the safety data collected as well.

**Results:**

Adverse events were observed in 198 (16.2) patients, among which severe AEs occurred in 16 (1.3%) patients. Meanwhile, bivalirudin-related ADRs were reported in 66 (5.4%) patients, among which 5 (0.4%) patients experienced bivalirudin-related severe ADRs. Besides, thrombocytopenia and bleeding occurred in 45 (3.7%) and 19 (1.5%) patients, respectively. The subsequent multivariate logistic analysis revealed that age >75 years [*p* = 0.017, odds ratio (*OR*) = 1.856] and spontaneous coronary artery dissection (SCAD) (*p* = 0.030, *OR* = 2.022) were independently related to higher ADR risk; SCAD (*p* = 0.017, *OR* = 2.426) was independently correlated with higher thrombocytopenia risk, while radial artery access (*p* = 0.015, *OR* = 0.352) was independently correlated with lower thrombocytopenia risk; and the administration of bivalirudin preoperatively or intraoperatively (*p* = 0.013, *OR* = 5.097) was independently associated with higher bleeding risk.

**Conclusion:**

Bivalirudin presents a favorable safety profile regarding ADRs, thrombocytopenia, and bleeding in Chinese patients with high bleeding risk undergoing PCI.

## Introduction

Percutaneous coronary intervention (PCI) is a commonly performed, minimally invasive treatment modality for cardiovascular diseases that ameliorates the stenosis of the coronary artery and thus improves the prognosis of patients with cardiovascular diseases (1, 2). Although patients who receive PCI may face in-stent restenosis, the administration of anticoagulants, such as unfractionated heparin (often combined with glycoprotein IIb/IIIa inhibitors) reduces the risk of in-stent restenosis, thus being recommended (3, 4). However, the administration of anticoagulants could induce several adverse events (AEs), such as thrombocytopenia and bleeding (5–7). Therefore, the choice of anticoagulants remains controversial in patients undergoing PCI, especially for those with high bleeding risks, such as higher age, or complicated with diabetes mellitus or renal function impairment (8, 9).

Bivalirudin is a direct thrombin inhibitor with multiple advantages, such as quick onset, short half-life, linear pharmacodynamic and pharmacokinetic properties, and less likely to induce bleeding (10, 11). Currently, a bunch of clinical trials have demonstrated that bivalirudin is a close equivalent to heparin regarding anticoagulation effects with an even better safety profile during PCI (12–18). Notably, it is also suggested that in patients with high bleeding risk, such as those with diabetes mellitus or chronic kidney diseases, bivalirudin induces fewer incidence of major bleeding (18–20). Therefore, bivalirudin might be a suitable approach for patients undergoing PCI with high bleeding risk. However, since its application in China in recent years, not much data are available regarding its safety profile in Chinese patients, not to mention those with high bleeding risk.

The present prospective, multi-center, intensive monitoring study focused on investigating the occurrence and risk factors for AEs and adverse drug reactions (ADRs), especially thrombocytopenia and bleeding in Chinese patients with high bleeding risk undergoing PCI with bivalirudin as an anticoagulant in a wide range of the population.

## Materials and Methods

### Patients

Between July 2018 and June 2019, a total of 3,049 patients who underwent PCI and received bivalirudin as an anticoagulant in 27 Chinese medical centers were enrolled in a prospective, multi-center, intensive monitoring study that aimed to evaluate the safety of bivalirudin in a wide range of population (summary of AEs and ADRs are shown in Supplementary Table 1). Out of 3,049 patients, 1,226 patients with high bleeding risk were analyzed in this study. The enrollment criteria were set as (a) patients whose ages were more than 18 years old; (b) patients who were about to receive PCI and bivalirudin; and (c) patients who had one of the following conditions were defined as patients with high bleeding risk: (i) age >75 years; (ii) diabetes mellitus; and (iii) renal function impairment (estimated glomerular filtration rate ranged from 30 to 60 ml/min) (21). The patients with PCI contraindications, hypersensitivity to bivalirudin, and pregnant or lactating patients were excluded from the study. During the process of the study, the therapy of all patients and their medication usage were determined by attending physicians on the basis of the actual clinical situation and were not interfered by the study. This study was approved by the Ethics Committee of each participating center with Beijing Anzhen Hospital Affiliated with Capital Medical University (approval number: KS2019012) and Peking University People’s Hospital (approval number: 2018PHA092-001) as the primary Ethics Committees. All patients provided written informed consent.

### Collection of Data

Clinical data were collected as follows: (i) demographic characteristics: age and gender; (ii) medical history: history of diabetes mellitus, allergy, critical respiratory disease, renal function impairment, and cardiac surgery; (iii) clinical presentation: unstable angina (UA); ST-segment elevation myocardial infarction (STEMI); non-ST-segment elevation myocardial infarction (NSTMI); and spontaneous coronary artery dissection (SCAD); and (iv) treatment characteristics: operative timing, types of coronary interventional therapy, arterial access, culprit vessel, administration of bivalirudin, and combined with glycoprotein (GP) IIb/IIIa inhibitors. Besides, safety data were also collected from all patients. AEs and ADRs were recorded particularly from hospital admission to 72 h after completion of bivalirudin administration. In addition, patients were followed up on the 30th day, and the data were also collected at that time. AEs and ADRs were classified by the Systematic Organ Classification (SOC) and Preferred Term (PT) of the International Conference on the Coordination of International Drug Registration (ICH) Medical Dictionary for Regulatory Activities (MedDRA) 23.0.

### Definitions

Adverse events were defined as any unfavorable and unintended sign (such as an abnormal laboratory finding), symptom, or disease temporally associated with the use of a medical treatment that may or may not be considered related to the medical treatment. ADRs were defined as the harmful reaction of qualified drugs that were irrelevant to the purpose of medication under normal usage and dosage. Severe adverse events (SAEs) and severe adverse drug reactions (SADRs) were defined as one of the following events: (i) leading to death; (ii) life-threatening consequences; (iii) leading to carcinogenesis, teratogenesis, and birth defects; (iv) leading to significant or permanent human disability or organ function damage; (v) resulting in hospitalization or prolonged length of stay; and (vi) leading to other important medical events, if not treated, the above listed conditions may occur. The severity of AEs and ADRs was divided into three levels according to the following criteria: (i) mild: symptoms were transient and did not affect the patient’s normal daily activities; (ii) moderate: symptoms were obvious to affect the patient’s normal daily activities, but tolerable, which did not require the patient to stop medication; and (iii) severe: symptoms were obvious, intolerable and affected the patient’s normal daily activities, which required the patient to stop the medication.

### Statistical Analysis

Continuous variables were expressed as mean ± SD, and categorical variables were expressed as numbers (percentage). The unknown data were not included in the analysis. Correlations of characteristics with ADRs were determined by the chi-square test and Fisher’s exact test. Logistic regression analyses for ADRs, thrombocytopenia, and bleeding were performed, and the forward stepwise methodology was applied for screening the independent prediction factors. A *p*-value of < 0.05 was considered statistically significant. All analyses were performed using SAS 9.4 (SAS Institute, Inc., Cary, NC, United States).

## Results

### Characteristics of Patients and Treatment

The detailed characteristics of patients and treatment are listed in Table 1 and Supplementary Table 2, respectively. In brief, the patients included 450 (36.7%) women and 776 (63.3%) men with a mean age of 70.9 ± 11.1 years. Meanwhile, the numbers of patients presented with UA, STEMI, NSTMI, SCAD, and others were 460 (37.5%), 426 (34.8%), 197 (16.1%), 140 (11.4%), and 3 (0.2%), respectively (Table 1). Regarding the treatment characteristics, 1,143 (93.2%), 77 (6.3%), 1 (0.1%), and 5 (0.4%) patients received PCI through the radial artery, femoral artery, brachial artery, and others, respectively. In addition, 49 (4.0%) patients were administrated with preoperative or intraoperative bivalirudin, 1,049 (85.6%) patients took bivalirudin within 4 h of operation, and 128 (10.4%) patients received bivalirudin after 4 h of operation. Moreover, 865 (70.6%) patients were administrated with bivalirudin combining GP IIb/IIIa inhibitors (Supplementary Table 2).

**TABLE 1:** Clinical characteristics.

Items	Patients (*N* = 1226)
**Demographic characteristics**	
Age (years), mean ± SD	70.9 ± 11.1
Gender, No. (%)	
Female	450 (36.7)
Male	776 (63.3)
**Medical history**	
History of diabetes mellitus, No. (%)	758 (61.8)
History of allergy, No. (%)	133 (10.8)
History of cardiac surgery, No. (%)	128 (10.4)
History of renal function impairment, No. (%)	97 (7.9)
History of critical respiratory disease, No. (%)	71 (5.8)
**Clinical presentation**	
UA, No. (%)	460 (37.5)
STEMI, No. (%)	426 (34.8)
NSTMI, No. (%)	197 (16.1)
SCAD, No. (%)	140 (11.4)
Others, No. (%)	3 (0.2)

*SD, standard deviation; UA, unstable angina; STEMI, ST-segment elevation myocardial infarction; NSTMI, non-ST-segment elevation myocardial infarction; SCAD, spontaneous coronary artery dissection.*

### Adverse Events, Adverse Drug Reactions, Thrombocytopenia, and Bleeding

**FIGURE 1:**
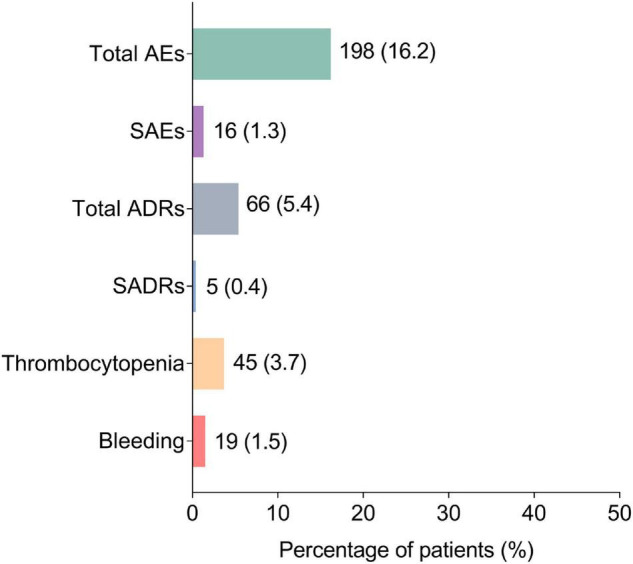
Occurrence of AEs, SAEs, ADRs, SADRs, thrombocytopenia, and bleeding. AEs, adverse events; SAEs, severe adverse events; ADRs, adverse drug reactions; SADRs, severe adverse drug reactions.

Generally, AEs occurred in 198 (16.2%) patients, among which SAEs were reported in 16 (1.3%) patients. Meanwhile, ADRs and SADRs were observed in 66 (5.4%) and 5 (0.4%) patients, respectively. Notably, thrombocytopenia occurred in 45 (3.7%) patients and bleeding was reported in 19 (1.5%) patients (Figure 1).

**FIGURE 2:**
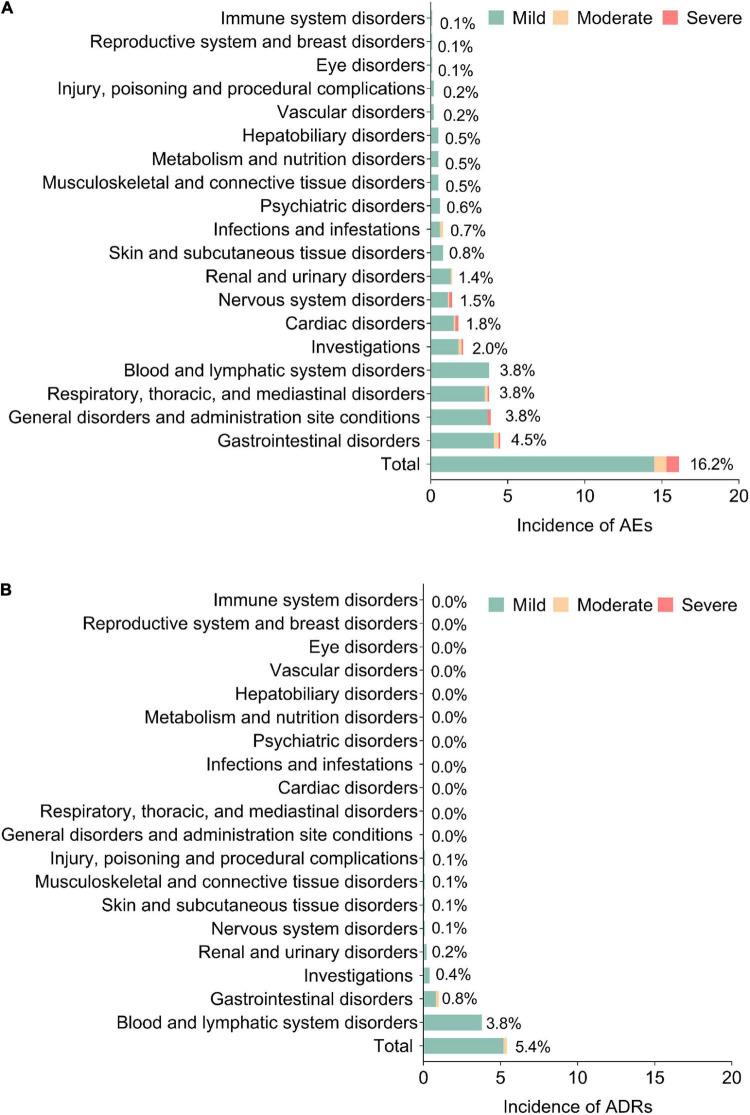
Detailed AEs and ADRs. Incidence of AEs **(A)** and ADRs **(B)** in system organ class (SOC). AEs, adverse events; ADRs, adverse drug reactions.

In detail, the majority of the AEs were mild (14.5%); the most commonly occurred AEs (by SOC) were gastrointestinal disorders (4.5%), general disorders and administration site conditions (3.8%), respiratory, thoracic, and mediastinal disorders (3.8%), as well as blood and lymphatic system disorders (3.8%) (Figure 2A). Meanwhile, the majority of ADRs were also mild (5.2%); the most commonly occurring ADRs were blood and lymphatic disorders (3.8%) and gastrointestinal disorders (0.8%) (Figure 2B).

### Factors Related to Adverse Drug Reactions

Correlation analyses revealed that age >75 years was correlated with a higher incidence of ADRs (*p* = 0.017), while other patient characteristics or treatment characteristics were not correlated with ADRs (all *p* > 0.05) (Table 2). Further univariate logistic regression analysis confirmed that age >75 years was correlated with a higher incidence of ADRs (*p* = 0.018, *OR* = 1.841); it also found that SCAD was associated with a higher incidence of ADRs (*p* = 0.033, *OR* = 1.995). Moreover, forward stepwise multivariate logistic regression analysis revealed that both age >75 years (*p* = 0.017, *OR* = 1.856) and SCAD (*p* = 0.030, *OR* = 2.022) were independently related to ADRs (Table 3).

**TABLE 2:** Correlations of characteristics with ADRs.

Items	ADRs		*P*-value
	
	No	Yes	
Age, No. (%)			**0.017**
>75 years	528 (45.5)	40 (60.6)	
≤75 years	632 (54.5)	26 (39.4)	
Gender, No. (%)			0.839
Male	735 (63.4)	41 (62.1)	
Female	425 (36.6)	25 (37.9)	
History of diabetes mellitus, No. (%)			0.834
Yes	718 (61.9)	40 (60.6)	
No	442 (38.1)	26 (39.4)	
History of allergy, No. (%)			0.948
Yes	126 (10.9)	7 (10.6)	
No	1034 (89.1)	59 (89.4)	
History of cardiac surgery, No. (%)			0.646
Yes	120 (10.3)	8 (12.1)	
No	1040 (89.7)	58 (87.9)	
History of renal function impairment, No. (%)			0.193
Yes	89 (7.7)	8 (12.1)	
No	1071 (92.3)	58 (87.9)	
History of critical respiratory disease, No. (%)			1.000
Yes	68 (5.9)	3 (4.5)	
No	1092 (94.1)	63 (95.5)	
Clinical presentation, No. (%)			0.250
UA	440 (37.9)	20 (30.3)	
STEMI	404 (34.9)	22 (33.3)	
NSTMI	186 (16.0)	11 (16.7)	
SCAD	127 (10.9)	13 (19.7)	
Others	3 (0.3)	0 (0.0)	
Operative timing, No. (%)			0.820
Elective operation	722 (62.2)	42 (63.6)	
Emergency operation	438 (37.8)	24 (36.4)	
Types of coronary interventional therapy, No. (%)			0.804
Stent implantation	1109 (95.6)	64 (97.0)	
Balloon dilatation	46 (4.0)	2 (3.0)	
Thrombus aspiration	0 (0.0)	0 (0.0)	
Others	5 (0.4)	0 (0.0)	
Arterial access, No. (%)			0.227
Radial artery	1085 (93.6)	58 (87.9)	
Femoral artery	69 (5.9)	8 (12.1)	
Brachial artery	1 (0.1)	0 (0.0)	
Others	5 (0.4)	0 (0.0)	
Culprit vessel, No. (%)			0.180
Single	876 (75.5)	45 (68.2)	
Multiple	284 (24.5)	21 (31.8)	
Administration of bivalirudin, No. (%)			0.523
Preoperative or intraoperative	45 (3.9)	4 (6.1)	
Post-operative ≤4 h	992 (85.5)	57 (86.3)	
Post-operative >4 h	123 (10.6)	5 (7.6)	
Combined with GP IIb/IIIa inhibitors, No. (%)			0.499
Yes	816 (70.3)	49 (74.2)	
No	344 (29.7)	17 (25.8)	

*The value of p < 0.05 was considered statistically significant. ADRs, adverse drug reactions; UA, unstable angina; STEMI, ST-segment elevation myocardial infarction; NSTMI, non-ST-segment elevation myocardial infarction; SCAD, spontaneous coronary artery dissection; GP, glycoprotein. Bolded values are representing items with statistical significance.*

**TABLE 3:** Logistic regression analysis for ADRs.

Items	*P*-value	OR	95% CI	
			
			Lower	Upper
**Univariate logistic regression analysis**				
Age >75 years	**0.018**	1.841	1.109	3.058
Gender (male)	0.839	0.948	0.569	1.582
History of diabetes mellitus	0.834	0.947	0.570	1.574
History of allergy	0.948	0.974	0.435	2.178
History of cardiac surgery	0.647	1.195	0.557	2.564
History of renal function impairment	0.197	1.660	0.768	3.585
History of critical respiratory disease	0.657	0.765	0.234	2.498
Clinical presentation (UA)	0.215	0.711	0.415	1.219
Clinical presentation (STEMI)	0.804	0.936	0.553	1.583
Clinical presentation (NSTMI)	0.892	1.047	0.538	2.039
Clinical presentation (SCAD)	**0.033**	1.995	1.058	3.761
Operative timing (elective operation)	0.820	1.062	0.634	1.777
Types of coronary interventional therapy (stent implantation)	0.598	1.472	0.350	6.181
Arterial access (radial artery)	0.081	0.501	0.231	1.088
Culprit vessel (multiple)	0.182	1.439	0.843	2.458
Administration of bivalirudin (preoperative or intraoperative)	0.383	1.599	0.557	4.587
Administration of bivalirudin (post-operative ≤4 h)	0.849	1.073	0.521	2.207
Administration of bivalirudin (post-operative >4 h)	0.436	0.691	0.272	1.753
Combined with GP IIb/IIIa inhibitors	0.500	1.215	0.690	2.140
Adequate administration of bivalirudin	0.183	1.668	0.785	3.545
**Forward stepwise multivariate logistic regression analysis**				
Age >75 years	**0.017**	1.856	1.117	3.085
Clinical presentation (SCAD)	**0.030**	2.022	1.070	3.822

*The value of p < 0.05 was considered statistically significant. ADRs, adverse drug reactions; OR, odds ratio; CI, confidence interval; UA, unstable angina; STEMI, ST-segment elevation myocardial infarction; NSTMI, non-ST-segment elevation myocardial infarction; SCAD, spontaneous coronary artery dissection; GP, glycoprotein.*

*Bolded values are representing items with statistical significance.*

### Risk Factors for Thrombocytopenia

Univariate logistic regression analysis revealed that SCAD (*p* = 0.024, *OR* = 2.310) was associated with a higher risk of thrombocytopenia, while PCI through the radial artery (*p* = 0.021, *OR* = 0.373) was correlated with a lower risk of thrombocytopenia. Further forward stepwise multivariate logistic regression analysis displayed that SCAD (*p* = 0.017, *OR* = 2.426) was an independent factor for higher risk of thrombocytopenia, while PCI through the radial artery (*p* = 0.015, *OR* = 0.352) was an independent factor for lower risk of thrombocytopenia (Table 4).

**TABLE 4:** Logistic regression analysis for thrombocytopenia.

Items	*P*-value	OR	95% CI	
			
			Lower	Upper
**Univariate logistic regression analysis**				
Age (>75 years)	0.064	1.774	0.967	3.257
Gender (male)	0.633	1.166	0.621	2.192
History of diabetes mellitus	0.234	0.696	0.383	1.265
History of allergy	0.586	1.277	0.530	3.075
History of cardiac surgery	0.881	1.075	0.417	2.775
History of renal function impairment	0.421	1.480	0.570	3.840
History of critical respiratory disease	0.317	0.361	0.049	2.657
Clinical presentation (UA)	0.782	0.916	0.492	1.705
Clinical presentation (STEMI)	0.249	0.674	0.344	1.318
Clinical presentation (NSTMI)	0.924	0.961	0.423	2.184
Clinical presentation (SCAD)	**0.024**	2.310	1.118	4.774
Operative timing (elective operation)	0.124	1.693	0.865	3.312
Types of coronary interventional therapy (stent implantation)	0.489	2.027	0.274	14.997
Arterial access (radial artery)	**0.021**	0.373	0.161	0.864
Culprit vessel (multiple)	0.184	1.536	0.815	2.895
Administration of bivalirudin (preoperative or intraoperative)	0.542	0.536	0.072	3.976
Administration of bivalirudin (post-operative ≤4 h)	0.287	1.759	0.622	4.973
Administration of bivalirudin (post-operative >4 h)	0.404	0.603	0.184	1.975
Combined with GP IIb/IIIa inhibitors	0.455	1.302	0.652	2.599
Adequate administration of bivalirudin	0.208	1.831	0.715	4.694
**Forward stepwise multivariate logistic regression analysis**				
Clinical presentation (SCAD)	**0.017**	2.426	1.168	5.039
Arterial access (radial artery)	**0.015**	0.352	0.151	0.818

*The value of p < 0.05 was considered statistically significant. OR, odds ratio; CI, confidence interval; UA, unstable angina; STEMI, ST-segment elevation myocardial infarction; NSTMI, non-ST-segment elevation myocardial infarction; SCAD, spontaneous coronary artery dissection; GP, glycoprotein. Bolded values are representing items with statistical significance.*

### Risk Factors for Bleeding

According to the univariate logistic regression analysis, administration of bivalirudin preoperatively or intraoperatively (*p* = 0.016, *OR* = 4.732) was associated with a higher risk of bleeding, but administration of bivalirudin within 4 h of operation (*p* = 0.040, *OR* = 0.358) was associated with a lower risk of bleeding. After adjusted by multivariate logistic regression, administration of bivalirudin preoperatively or intraoperatively (*p* = 0.013, *OR* = 5.097) was an independent risk factor for bleeding (Table 5).

**TABLE 5:** Logistic regression analysis for bleeding.

Items	*P*-value	OR	95% CI	
			
			Lower	Upper
**Univariate logistic regression analysis**				
Age (>75 years)	0.313	1.605	0.641	4.017
Gender (male)	0.061	0.416	0.166	1.041
History of diabetes mellitus	0.133	2.342	0.773	7.099
History of allergy	0.442	0.452	0.060	3.417
History of cardiac surgery	0.990	1.009	0.231	4.419
History of renal function impairment	0.672	1.377	0.313	6.050
History of critical respiratory disease	0.921	0.902	0.119	6.858
Clinical presentation (UA)	0.062	0.308	0.089	1.062
Clinical presentation (STEMI)	0.250	1.705	0.687	4.229
Clinical presentation (NSTMI)	0.973	0.979	0.283	3.392
Clinical presentation (SCAD)	0.193	2.100	0.687	6.419
Operative timing (elective operation)	0.075	0.434	0.173	1.087
Types of coronary interventional therapy (stent implantation)	0.839	0.810	0.106	6.187
Arterial access (radial artery)	0.793	1.312	0.173	9.951
Culprit vessel (multiple)	0.884	1.080	0.386	3.023
Administration of bivalirudin (preoperative or intraoperative)	**0.016**	4.732	1.332	16.815
Administration of bivalirudin (post-operative ≤4 h)	**0.040**	0.358	0.134	0.954
Administration of bivalirudin (post-operative >4 h)	0.447	1.623	0.466	5.648
Combined with GP IIb/IIIa inhibitors	0.422	1.575	0.519	4.778
Adequate administration of bivalirudin	0.760	0.841	0.276	2.557
**Forward stepwise multivariate logistic regression analysis**				
Gender (male)	0.051	0.399	0.158	1.004
Administration of bivalirudin (preoperative or intraoperative)	**0.013**	5.097	1.420	18.296

*The value of p < 0.05 was considered statistically significant. OR, odds ratio; CI, confidence interval; UA, unstable angina; STEMI, ST-segment elevation myocardial infarction; NSTMI, non-ST-segment elevation myocardial infarction; SCAD, spontaneous coronary artery dissection; GP, glycoprotein. Bolded values are representing items with statistical significance.*

## Discussion

In the present real-world study observing the safety profile of bivalirudin as an anticoagulant in Chinese patients undergoing PCI with high bleeding risk, the results revealed that the incidences of AEs, SAEs, bivalirudin-related ADRs, and SADRs were 16.2, 1.3, 5.4, and 0.4%, respectively. Meanwhile, thrombocytopenia and bleeding occurred in 3.7 and 1.5% of patients, respectively. Besides, older age and SCAD were independent factors for the higher risk of bivalirudin-related ADRs; SCAD was an independent factor for the higher risk of thrombocytopenia, while radial artery access was an independent factor for the lower risk of thrombocytopenia; preoperative or intraoperative administration of bivalirudin was an independent factor for the higher risk of bleeding.

Bivalirudin is considered as an alternative option for heparin-based anticoagulants in patients undergoing PCI. So far, a number of clinical trials have been conducted and revealed the similarity or even superiority of bivalirudin against heparin regarding the safety profile (12–18). For instance, a recent trial recruiting 6,006 myocardial infarction patients undergoing PCI reveals that the incidences of major bleeding (2.8 vs. 2.8%) and death of any cause (2.9 vs. 2.8%) are similar in patients who receive bivalirudin or heparin monotherapy during PCI (12). Meanwhile, the BRIGHT trial discloses that bivalirudin reduces the 30-day bleeding rate (4.1%) compared with heparin monotherapy (7.5%) or heparin plus tirofiban (12.3%) in acute myocardial infarction patients undergoing PCI (13). Moreover, a pre-specified analysis from the EUROMAX trial shows that bivalirudin reduces the risk of mortality (*OR* = 0.53, 95% *CI*: 0.33–0.87) or major bleeding (*OR* = 0.44, 95% *CI* 0.24–0.82) compared with heparin plus bailout glycoprotein IIb/IIIa inhibitors in patients with STEMI undergoing PCI (14). Apart from that, considering that patients at a higher bleeding risk may benefit from bivalirudin (22, 23), studies have been performed to verify this issue. For example, a meta-analysis reviewing REPLACE-2, ACUITY, HORIZONS-AMI, ISAR-REACT-4, and EUROMAX trials found that bivalirudin decreases major bleeding in STEMI patients with impairment of renal function undergoing PCI (24). Furthermore, another meta-analysis reviewing REPLACE-2, ACUITY, and HORIZONS-AMI trials reveals that in patients with diabetes mellitus undergoing PCI, bivalirudin also reduces major bleeding compared with heparin plus glycoprotein IIb/IIIa inhibitors (4.3 vs. 6.6%) (20). In addition, similar results are also concluded in older adult patients undergoing PCI (19). Currently, a loading dose of 0.10 mg/kg, followed by 0.25 mg/kg/day of bivalirudin is recommended by the American Heart Association for patients undergoing PCI (25). However, since bivalirudin is recently applied in China, little data could be acquired regarding the safety profile of bivalirudin as anticoagulant in Chinese patients with high bleeding risk undergoing PCI.

In the present study that observes the safety profile of bivalirudin in Chinese patients with high bleeding risk undergoing PCI, the data revealed that the incidences of AEs and SAEs bivalirudin-related ADRs and SADRs were 16.2, 1.3, 5.4, and 0.4%, respectively. Meanwhile, the most commonly occurred AEs (by SOC) were gastrointestinal disorders (4.5%), general disorders, and administration site conditions (3.8%), respiratory, thoracic, and mediastinal disorders (3.8%), as well as blood and lymphatic system disorders (3.8%); and the most commonly occurred ADRs were blood and lymphatic disorders (3.8%) and gastrointestinal disorders (0.8%). The incidence of AEs in the current study was within the range of previous reports (13, 26, 27). However, since the incidence of bivalirudin-related ADRs is rarely reported, it was unable to compare these data with other studies. Meanwhile, the incidence of bleeding was lower compared with previous reports (12, 13), which could be a result of the differences in the follow-up period, PCI characteristics, bivalirudin administration, etc. between the present study and previous studies. In the current study, four patients died due to severe AEs, among which the death of three patients was irrelevant to bivalirudin administration, while the death of the other patient was unable to be assessed. Besides, no difference of ADRs was found in patients undergoing elective PCI or emergency PCI, indicating that bivalirudin might be used under both circumstances. In the current study, “post-operative ≤4 h” and “post-operative >4 h” refers to the fact that, patients received an initial dose of 0.75 mg/kg, followed by 1.75 mg/kg/h until ≤4 h after PCI, and >4 h after PCI, respectively. Our data showed that no difference in ADRs was found in patients receiving different administration of bivalirudin, thus it could be concluded that bivalirudin dosage increase/decrease would not affect the incidence of ADRs, which could be adjusted dependent on patients’ conditions.

Furthermore, the present study investigated risk factors for bivalirudin-related ADRs, thrombocytopenia, and bleeding, which revealed that higher age and SCAD were independent factors for higher risk of bivalirudin-related ADRs; SCAD was an independent factor for higher risk of thrombocytopenia, while radial artery access was an independent factor for a lower risk of thrombocytopenia. Possible explanations might be that: (1) patients of higher age had worse body conditions, thus correlated with higher ADRs; (2) although carefully selected in patients, those with clinical presentation of SCAD treated with PCI still might face with a higher risk of Iatrogenic dissection and hematoma extending (28), thereby correlated with a higher risk of ADRs and thrombocytopenia; and (3) radial artery access is associated with a lower risk of bleeding (29, 30), thus bivalirudin dosage was reduced in these patients, resulting in the lower risk of thrombocytopenia. Moreover, preoperative or intraoperative administration of bivalirudin was an independent factor for increased risk of bleeding, raising the hypothesis that post-operative administration of bivalirudin may be a more appropriate approach for patients with high bleeding risk undergoing PCI, while further trials should be conducted to verify this.

It could not be denied that several limitations existed in this study. First, although the sample size of this study was relatively large, the incidences of bivalirudin-related SADRs, thrombocytopenia, and bleeding were relatively low, resulting in lower statistical reliability. Second, the interaction of administration time of bivalirudin with bleeding was not the primary objective of this study, which could be verified in further randomized controlled trials. Third, the anticoagulant efficacy of bivalirudin should be observed in a large population in the future. Fourth, the definition of high bleeding risk was referred to a previous study (21), while other definitions of high bleeding risk should be considered in the future to further verify the safety of bivalirudin. Fifth, this is an observational registry study focusing on bivalirudin, further studies with a control cohort should be conducted for the verification of the safety of bivalirudin.

Collectively, bivalirudin presents a favorable safety profile regarding ADRs, thrombocytopenia, and bleeding in Chinese patients with high bleeding risk undergoing PCI.

## Data Availability Statement

The original contributions presented in the study are included in the article/Supplementary Material, further inquiries can be directed to the corresponding authors.

## Ethics Statement

The studies involving human participants were reviewed and approved by The First Affiliated Hospital of Nanchang University; Xuzhou Central Hospital of Jiangsu Province; Tai’an First People’s Hospital; Puyang Oilfield General Hospital; Wuzhou People’s Hospital; Mianyang Central Hospital; and Tangshan Worker’s Hospital. The patients/participants provided their written informed consent to participate in this study.

## Author Contributions

XP, ZheL, CL, and ZJ made substantial contributions to the design of the present study. XP, ZheL, DL, ZhoL, ZhaL, CL, and ZJ performed data acquisition and interpretation. XP, ZheL, DL, ZhoL, and ZhaL drafted the work. CL and ZJ critically revised the manuscript for important intellectual content. All authors approved the final version of the manuscript and agreed to be accountable for all aspects of the work in ensuring that questions related to the accuracy or integrity of the work are appropriately investigated and resolved.

## Conflict of Interest

The authors declare that the research was conducted in the absence of any commercial or financial relationships that could be construed as a potential conflict of interest.

## Publisher’s Note

All claims expressed in this article are solely those of the authors and do not necessarily represent those of their affiliated organizations, or those of the publisher, the editors and the reviewers. Any product that may be evaluated in this article, or claim that may be made by its manufacturer, is not guaranteed or endorsed by the publisher.
