# Supplementary material for: Bivalirudin Presents a Favorable Safety Profile Regarding Adverse Drug Reactions, Thrombocytopenia, and Bleeding in Chinese Patients With High Bleeding Risk Undergoing Percutaneous Coronary Intervention: A Prospective, Multi-Center, Intensive Monitoring Study
Source: Front Cardiovasc Med. 2022 Jun 16;9:821322. doi: 10.3389/fcvm.2022.821322 (PMC10166107; doi:10.3389/fcvm.2022.821322)
Supplement: Supplementary file 1 [file Table_1.docx]

**Supplementary table 1.** Summary of AEs and ADRs

| Items | Number of times | Incidence, No. (%) | 95% CI of incidence |
| --- | --- | --- | --- |
| **Adverse events (AEs)** |  |  |  |
| Total AEs | 829 | 414 (13.58) | 12.38-14.84 |
| SAEs | 38 | 31 (1.02) | 0.69-1.44 |
| Death due to AEs | 9 | 8 (0.26) | 0.11-0.52 |
| **Adverse drug reactions (ADRs)** |  |  |  |
| Total ADRs | 130 | 118 (3.87) | 3.21-4.62 |
| SADRs | 7 | 7 (0.23) | 0.09-0.47 |
| Death due to ADRs | 0 | 0 (0.00) | - |
| New ADRs | 8 | 7 (0.23) | 0.09-0.47 |
| Bleeding | 37 | 34 (1.12) | 0.77-1.55 |
| BARC type 0 | 1 | 1 (0.03) | 0.00-0.18 |
| BARC type 1 | 29 | 27 (0.89) | 0.58-1.29 |
| BARC type 2 | 2 | 2 (0.07) | 0.01-0.24 |
| BARC type 3a | 5 | 4 (0.13) | 0.04-0.34 |
| Thrombocytopenia | 79 | 79 (2.59) | 2.06-3.22 |

AEs, adverse events; SAEs, severe adverse events; ADRs, adverse drug reactions; SADRs, severe adverse drug reactions; BARC, Bleeding Academic Research Consortium; CI, confidence interval.
