# Supplementary material for: Bivalirudin Presents a Favorable Safety Profile Regarding Adverse Drug Reactions, Thrombocytopenia, and Bleeding in Chinese Patients With High Bleeding Risk Undergoing Percutaneous Coronary Intervention: A Prospective, Multi-Center, Intensive Monitoring Study
Source: Front Cardiovasc Med. 2022 Jun 16;9:821322. doi: 10.3389/fcvm.2022.821322 (PMC10166107; doi:10.3389/fcvm.2022.821322)
Supplement: Supplementary file 2 [file Table_2.docx]

**Supplementary Table 2.** Characteristics of treatment

| Items | Patients (N = 1226) |
| --- | --- |
| Operative timing, No. (%) |  |
| Emergency operation | 462 (37.7) |
| Elective operation | 764 (62.3) |
| Types of coronary interventional therapy, No. (%) |  |
| Stent implantation | 1173 (95.7) |
| Balloon dilatation | 48 (3.9) |
| Thrombus aspiration | 0 (0.0) |
| Others | 5 (0.4) |
| Arterial access, No. (%) |  |
| Radial artery | 1143 (93.2) |
| Femoral artery | 77 (6.3) |
| Brachial artery | 1 (0.1) |
| Others | 5 (0.4) |
| Culprit vessel, No. (%) |  |
| Single | 921 (75.1) |
| Multiple | 305 (24.9) |
| Administration of bivalirudin, No. (%) |  |
| Preoperative or intraoperative | 49 (4.0) |
| Postoperative ≤ 4h | 1049 (85.6) |
| Postoperative > 4h | 128 (10.4) |
| Combined with GP IIb/IIIa inhibitors, No. (%) | 865 (70.6) |

GP, glycoprotei.
